# Who will benefit from computerized cognitive remediation therapy? Evidence from a multisite randomized controlled study in schizophrenia

**DOI:** 10.1017/S0033291719001594

**Published:** 2020-07

**Authors:** Shuping Tan, Xiaolin Zhu, Hongzhen Fan, Yunlong Tan, Fude Yang, Zhiren Wang, Yanli Zhao, Fengmei Fan, Junhua Guo, Zhanjiang Li, Wenxiang Quan, Xiangqun Wang, Clare Reeder, Dongfeng Zhou, Yizhuang Zou, Til Wykes

**Affiliations:** 1 Beijing HuiLongGuan Hospital, Peking University HuiLongGuan Clinical Medical School, Beijing 100096, P.R. China; 2 Beijing Anding Hospital of Capital Medical University, Beijing 100088, P.R. China; 3 Institute of Mental Health, Peking University, Beijing 100191, P.R. China; 4 Department of Psychology, Institute of Psychiatry, Psychology and Neuroscience, King's College London, De Crespigny Park, London SE5 8AF, UK; 5 South London and Maudsley NHS Foundation Trust

**Keywords:** Cognitive deficits, computerized cognitive remediation therapy, schizophrenia

## Abstract

**Background:**

Computerized cognitive remediation therapy (CCRT) is generally effective for the cognitive deficits of schizophrenia. However, there is much uncertainty about what factors mediate or moderate effectiveness and are therefore important to personalize treatment and boost its effects.

**Method:**

In total, 311 Chinese inpatients with Diagnostic and Statistical Manual of Mental Disorders-IV schizophrenia were randomized to receive CCRT or Active control for 12 weeks with four to five sessions per week. All participants were assessed at baseline, post-treatment and 3-month follow-up. The outcomes were cognition, clinical symptoms and functional outcomes.

**Results:**

There was a significant benefit in the MATRICS Consensus Cognitive Battery (MCCB) total score for CCRT (*F*_1,258_ = 5.62; *p* = 0.02; effect size was 0.27, 95% confidence interval 0.04–0.49). There were no specific moderators of CCRT improvements. However, across both groups, Wisconsin Card Sort Test improvement mediated a positive effect on functional capacity and Digit Span benefit mediated decreases in positive symptoms. In exploratory analyses younger and older participants showed cognitive improvements but on different tests (younger on Symbol Coding Test, while older on the Spatial Span Test). Only the older age group showed MSCEIT benefits at post-treatment. In addition, cognition at baseline negatively correlated with cognitive improvement and those whose MCCB baseline total score was around 31 seem to derive the most benefit.

**Conclusions:**

CCRT can improve the cognitive function of patients with schizophrenia. Changes in cognitive outcomes also contributed to improvements in functional outcomes either directly or solely in the context of CCRT. Age and the basic cognitive level of the participants seem to affect the cognitive benefits from CCRT.

## Introduction

Cognitive impairment in schizophrenia is common and accounts for significant variation in real-world outcomes such as work performance even when supportive recovery programs are provided (Green *et al*., 2000; Bell *et al*., 2007). These impairments constitute a key component of recovery and are therefore logical treatment targets. They are resistant to current pharmacological treatments (Choi *et al*., 2013) and so a variety of cognitive remediation techniques have been developed to improve cognitive function in schizophrenia.

Cognitive remediation therapy (CRT) has moderate to large effects on cognitive outcomes (attention, memory, executive function, social cognition or metacognition) (Wykes *et al*., 2011) across different presentation modes e.g. paper and pencil (Wykes *et al*., 2007*a*, 2007*b*; Cella *et al*., 2014) or computer (Kurtz *et al*., 2015). The majority of reviews and meta-analyses have confirmed CRT benefits for both cognition and functioning in psychosis (Wykes *et al*., 2011; Tan and Liu, 2016). However, a few studies have found no benefits (Dickinson *et al*., 2010; Rass *et al*., 2012; Gomar *et al*., 2015). These rare instances might be explained by ineffective therapy or sampling differences, for instance including older participants as age has been shown to affect benefits (e.g. Wykes *et al*., 2009). As health resources are limited, it is crucial to identify whether some participants benefit more (or less) from CRT in order that scarce resources can be deployed efficiently. Previous research has suggested that age and baseline cognitive performance may moderate the impact of cognition (Wykes *et al*., 2009; Kontis *et al*., 2013; Ramsay *et al*., 2018). Previous studies feature small sample sizes that are insufficient for subgroup analysis. In the current study, a large sample of Chinese inpatients was recruited to investigate the efficacy of a new Chinese cognitive remediation computerized program, Computerized Cognitive Remediation Therapy (CCRT) for cognitive performance and then investigate whether these improvements contribute to improved functional capacity or symptoms. The size of the study allows the exploration of proposed moderators and mediators (age, cognition) of treatment benefit, as well as testing the CRT therapeutic model of improvements in cognition having an impact on functioning. Identifying predictors could guide personalization and tailored care and increase the benefits of cognitive remediation.

## Methods

### Design

This is a longitudinal, randomized, single blind multisite clinical trial which was part of a larger study. All participants who consented and fulfilled the inclusion criteria were randomly allocated to CCRT or Active control group in two hospitals (Beijing Anding Hospital and the Peking University Sixth Hospital) and at Beijing Huilongguan Hospital they were randomly allocated to three conditions (CCRT, Active control and Paper and pencil cognitive remediation). In this report only the comparisons between CCRT and Active control are presented. The primary cognitive outcome was MATRICS Consensus Cognitive Battery (MCCB) total score assessed at baseline (week 0), post-treatment (week 12) and follow-up (week 24). Secondary outcomes (cognition, functional capacity and symptoms) were assessed at the same times. All participants provided written informed consent, and the protocol was approved by the Beijing Huilongguan Hospital Ethics Committee (2006-3). The trial is registered at Chinese Clinical Trials Registry, identifier ChiCTR-TRC-08000249.

### Participants

In total, 311 voluntarily admitted in-patients with schizophrenia were recruited from 14 May 2007 to 1 March 2009 from Beijing Huilongguan Hospital, Beijing Anding Hospital and the Peking University Sixth Hospital. The final follow-up visit was on 13 October 2009. They had chronic schizophrenia and symptoms that required prolonged hospitalization but were clinically stable during the study period. The inclusion criteria were: Diagnostic and Statistical Manual, 4th ed (DSM-IV) (American Psychiatric Association, 1994) schizophrenia diagnosis confirmed by two psychiatrists; age 20–60 years; cognitive impairment [<4 categories on the Wisconsin Card Sort Test (WCST), or <7 on the WAIS-R Digit Span Backward test], 6+ years full-education (ensured full understanding of the task instructions), a stable dose and type of medication (for at least 1 month prior to inclusion and no anticipated medication change over the course of the study). Exclusion criteria included a planned medication change, any difficulty in communicating effectively with therapists, diagnosis of substance abuse as defined by the DSM-IV and a history of organic brain disorder or other severe organic disorder.

**Fig. 1.**
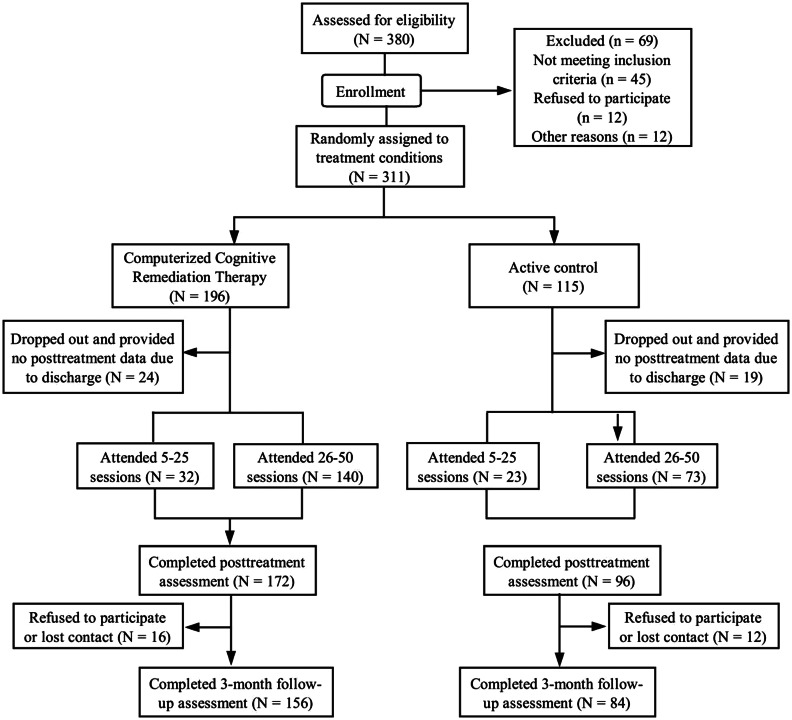
Treatment study flowchart.

The CONSORT flow diagram in Fig. 1 shows 311 people were finally randomized (CCRT, *N* = 196; Active control, *N* = 115). There were 198 inpatients from Beijing Huilongguan Hospital, 72 from Beijing Anding Hospital and 41 from the Peking University Sixth Hospital. The participants from Beijing Huilongguan hospital were randomized by 3:1 (CCRT *v.* Active control) and from the other two hospitals were randomized 1:1 (CCRT *v.* Active control) due to the limited numbers. Randomization was independently conducted by a psychiatrist not otherwise involved in the study at the completion of all baseline assessments. A random number table was used to generate randomization which was provided in sealed envelopes. As expected from random allocation, the two groups were well balanced in age, gender, education, duration of illness, cognitive difficulties and social behavior deficits. The dropout rates from the study were 12.2% and 16.5% (CCRT *v.* Active control) at post-treatment, and 20.4% and 26.9% (CCRT *v.* Active control) at follow-up.

### Outcome measures

#### Primary and secondary cognitive outcomes

Cognition was assessed using the Chinese version of the MCCB (Zou *et al*., 2009) which consists of 10 tests with seven domains and the alternate form was used at post-treatment. The primary outcome was the MCCB total score transformed to *T* scores (mean = 50, s.d. = 10) and the individual test scores were the secondary outcomes also transformed to *T* scores. The seven domains were:

(1) Speed of processing: Category Fluency Test, Trail Making Test-A, Symbol Coding Test.
(2) Attention/Vigilance: Continuous Performance Test (Continuous Performance Test-Identical Pair, CPT-IP).
(3) Working memory: Spatial Span Test (Wechsler Memory Scale-Third Edition, WMS-III); Digit Sequencing Test.
(4) Verbal learning: Hopkins Verbal Learning Test-Revised (HVLT, Chinese version).
(5) Visual learning: Brief Visuospatial Memory Test-Revised (BVMT-R).
(6) Reasoning and problem solving: Mazes Test (Neuropsychological Assessment Battery-Mazes, NAB-MAZES).
(7) Social cognition: Mayer-Salovery-Caruso Emotional Intelligence Test (MSCEIT, Chinese version): Managing Emotions (Caruso *et al*., 2002).

Other secondary cognitive outcomes included: Verbal Working memory: Wechsler Adult Intelligence Scale-III Digit Span Test with the key measure being the total score. Executive function was measured with the WCST (Heaton *et al*., 1993), and the key score was the number of completed categories.

#### Secondary symptom and function outcomes

Functional capacity was measured with a Chinese version (Cui *et al*., 2012) of UCSD Performance-based Skills Assessment (UPSA), which uses structured role-play scenarios to measure ability in two everyday living domains (Finance, e.g. making change; Communication, e.g. using the telephone for emergencies). The Finance task consists of 10 items; the Communication task has nine items. These tasks require about 8 and 5 min, respectively, to complete with the highest scores being 10 and 9.

A Chinese version (Li, 1987) of Nurse's Observation Scale for Inpatient Evaluation (NOSIE)-30 (Honigfeld *et al*., 1966) was used to evaluate behavior and functional capacity. The outcomes included were the total score, positive factor score (Social Competence, Social Interest, Personal Neatness) and negative factor score (Irritability, Manifest Psychosis, Retardation and Depression).

The Rosenberg self-esteem scale (Rosenberg, 1965) which had been translated into Chinese (Wang Ping *et al*., 1998) was used to measure global feelings of self-worth or self-acceptance from 10 items. The key outcome is the overall score with higher scores reflecting higher levels of self-esteem.

### Clinical assessment

The Chinese version of Positive and Negative Syndrome Scale (PANSS) (Yanlin and MingYuan, 2000), which has similar psychometric properties to those obtained in the original version (Kay, 1990; Tianmei *et al*., 2004), was used for the symptom assessment (PANSS total score, PANSS Positive score and PANSS Negative score).

### Data collection

All cognitive assessments were completed by four trained clinical psychologists who had at least 5 years' experience of psychometric testing (before baseline, the raters received a high consistent *κ* of 0.90). The clinical symptom rating (PANSS) was conducted by eight attending psychiatrists (before the study start, they achieved a satisfactory *κ* of 0.80). The functional capacity assessment was completed by four senior nurses who had at least 5 years' experience of psychiatry nursing (before assessment, these raters were trained and achieved a *κ* value of 0.85). All raters were blind to group assignment.

### Therapies

Both therapies were provided in groups and the duration and frequency of sessions were identical.

#### Cognitive remediation

Computer software (Computerized Cognitive Remediation Therapy System; CCRT) was developed from principles in Wykes and Reeder (Wykes and Reeder, 2005). It consists of 30 exercises that dynamically change in difficulty as accuracy reaches 80%. CCRT treatment was provided in four to five 45-min sessions per week over 12 weeks with a total of 50 sessions. It is supervised by experienced therapists at a ratio of 1 therapist to 4 participants. The therapist teaches participants to use CCRT in the first 2 weeks, and subsequent treatment is mainly carried out by the participant alone.

#### Active control

This therapy had the same number of sessions as CCRT but contained two different activities: learning to play a fairly easy instrument, usually the xylophone, and learning to dance, which were generally offered to almost every inpatient in these hospitals. The role of the therapist was to encourage these activities and take part actively in each session.

### Statistical analyses

All data were analyzed using IBM SPSS 20.0 (IBM Corp, Armonk, NY, USA).

#### Efficacy analyses

First, a series of intent-to-treat analyses were used to examine all outcome variables. General linear mixed modeling (GLM) with model parameters estimated by maximum likelihood based on normality was used for both the efficacy analysis and to explore secondary outcomes. Models included fixed effects for the experimental factors [main effect of group (CCRT or Active control) and time (post-therapy or follow-up) and a group × time interaction] and baseline values of the outcome measure was a covariate. A main effect of group would be interpreted as an effect of CCRT consistent across both post-treatment and follow-up. A significant group × time interaction term implies a differential effect of treatment at the post-treatment and follow-up, and then further simple effect analysis for the significant group × time interaction was conducted with Bonferroni correction for multiple comparisons. In addition, random effects for participants and study sites were included. We will examine predictors of drop-out and if any are significant we will incorporate them into all analyses.

#### Mediation and moderator analyses

CCRT improves functional outcome mainly by changing cognitive function and then this benefits functioning. CCRT can also promote functional improvement by enhancing the relationship between cognitive change and functional improvement. Cognitive improvement is therefore the mediator which is moderated by CCRT. For example, cognitive change was predictive of functional change only in the CR group (Reeder *et al*., 2004; Spaulding *et al*., 1999*a*, 1999*b*). In order to investigate these putative effects, analyses of covariance (ANCOVAs) were carried out with the follow-up measure of functional capacity as the dependent measure, cognitive change (post-treatment – baseline), group and an interaction between cognitive change and group as explanatory variables, and initial baseline level of functional capacity as a covariate. The interaction indicates that cognitive improvements could affect functional outcome if they were achieved in the context of CCRT. The model was re-fitted excluding the interaction term if it was not significant. A statistically significant and positive main effect of cognitive change on functional capacity and evidence of cognitive change improved by CCRT indicate that cognition could mediate functional improvement in CCRT.

In order to investigate the putative effects of MCCB baseline total score on cognitive benefits we performed a Pearson or partial correlation analysis controlling for age separately for the CCRT and the Active control groups, locally weighted scatterplot smoothing (LOESS) and polynomial fitting (quadratic polynomial). The MCCB difference (post-treatment – baseline) was considered as the cognitive benefit. The LOESS method provided an exploratory insight between two variables and help us choose parametric models (Cleveland and Devlin, 1988), and further polynomial fitting was used to clarify the model (Johnson, 1991).

In order to explore the influence of age on cognitive benefits, we divided the participants into two age groups (younger group = < or =39 years; older group >39 years). This cut-off was chosen because previous studies have shown differences for under and over 40 years (McGurk and Mueser, 2008; Wykes *et al*., 2009; Kontis *et al*., 2013). The GLM analyses were also used but these models included additional fixed effects for age group and two-way or three-way interaction for the moderator analyses. The model was re-fitted excluding the interaction term if it was not significant. Further simple effect analysis for the significant group × age group interaction was conducted.

Statistical thresholds for GLM analysis used *p* < 0.05. Because we are investigating potential moderators (age and MCCB baseline score) of cognitive benefits after CCRT treatment, we will adopt a broad view of significance with any group by X interaction below 0.1 as an effect to be investigated. In addition to the effects on the main cognitive outcome we will also carry out exploratory analyses on individual tests as others have indicated differential improvements by age (Thomas *et al*., 2017).

### Sample size and power of the study

The sample calculation assumes a standardized benefit of 0.49 (Twamley *et al*., 2003). Assuming a CCRT group of 108 people and an Active control group of 54 people the study would have 90% power to detect this difference based on an independent samples *t* test for unequal groups at the 5% significance level. This was increased to more than 130 people for the CCRT group and more than 65 people for the Active control group in order to account for possible dropout.

## Results

Table 1 shows the baseline characteristics of the randomized groups. There were no significant differences between the groups on demographics, antipsychotic type or chlorpromazine equivalents. Mean baseline scores of cognitive functions, clinical symptoms and functional outcomes were also similar between the two groups (see Table 2). No baseline characteristics predicted dropout and so none were added to our analyses (see online Supplementary Table S1 for details).

**Table 1.** Demographic, clinical and cognitive variables of the two groups

Characteristics	CCRT group (*n* = 196)	Active control group (*n* = 115)	*t*/χ^2^	*p* value
Sex (male/female)	122:74	69:46	0.154	0.695^a^
Mean age (s.d.), year	45.60 ± 8.68	44.24 ± 8.31	1.354	0.177^b^
Mean education years (s.d.), year	11.74 ± 2.82	11.86 ± 2.72	−0.370	0.711^b^
Marriage (yes/no)	48:148	28:87	0.001	0.978^a^
Duration of illness (s.d.), year	21.16 ± 9.77	19.18 ± 9.44	1.659	0.098^b^
Mean onset age (s.d.), year	24.44 ± 6.99	24.55 ± 7.10	−0.124	0.902^b^
MCCB total score	40.19 ± 10.62	40.36 ± 10.09	−0.140	0.889^b^
UPSA score	41.39 ± 9.41	38.36 ± 9.02	1.688	0.094^b^
Mean (s.d.) PANSS score				
Total score	59.77 ± 12.44	61.62 ± 13.43	−1.166	0.245^b^
Positive score	12.12 ± 4.76	12.39 ± 5.08	−0.454	0.650^b^
Negative score	17.02 ± 5.17	17.72 ± 6.10	−1.020	0.309^b^
Dose of chlorpromazine equivalents	407.39 ± 260.44	363.91 ± 221.15	1.408	0.160^b^
Psychiatric medication (typical/atypical and both)	31:147	16:85	0.114	0.736^a^

CCRT, Computerized Cognitive Remediation Therapy; MCCB, MATRICS Consensus Cognitive Battery; UPSA, UCSD Performance-Based Skills Assessment; PANSS, Positive and Negative Syndrome Scale.

a χ^2^ tests.

b Independent samples *t* test.

**Table 2.** Linear model for mean (s.d.) scores on cognitive function, clinical symptom and functional outcome by group (CCRT and Active control) for baseline, posttreatment and the 3 months follow-up

	CCRT group (*n* = 196/172/156)			Active control group (*n* = 115/96/84)		
Cognitive outcome	Baseline	Posttreatment	Follow-up	Baseline	Posttreatment	Follow-up
MCCB						
Speed of processing						
Category Fluency Test (animal)	47.27 (11.09)	49.31 (10.67)	49.28 (11)	46.39 (12.21)	47.44 (12.61)	48.47 (12.95)
Trail Making Test, Part A	41.28 (13.50)	42.79 (12.61)	43.60 (13.27)	42.81 (12.70)	44.22 (13.47)	47.38 (14.47)
Symbol Coding Test	40.93 (9.28)	41.75 (8.81)	43.43 (9.03)	40.35 (8.95)	40.04 (9.96)	43.1 (10.16)
Attention/Vigilance						
Continuous Performance Test	44.37 (9.57)	45.49 (10.36)	46.96 (10.73)	42.12 (9.28)	43.67 (9.37)	44.86 (11.12)
Working memory						
Digit Sequencing Test	45.17 (10.05)	46.83 (10.39)	47.26 (9.71)	43.42 (10.39)	45.86 (9.73)	45.42 (11.37)
Spatial Span Test	43.37 (11.63)	46.81 (10.70)	46.28 (12.67)	41.80 (12.70)	42.27 (11.11)	43.24 (12.55)
Verbal learning						
HVLT-R	42.93 (11.78)	43.60 (12.07)	46.95 (12.41)	42.71 (11.45)	41.22 (12.61)	44.9 (13.41)
Visual learning						
BVMT-R	43.22 (11.02)	46.82 (11.44)	47.40 (11.52)	44.33 (11.30)	46.99 (10.87)	46.35 (11.53)
Reasoning and problem solving						
Mazes Test	40.47 (13.15)	44.05 (11.61)	43.95 (11.20)	44.62 (12.65)	44.78 (12.50)	45.04 (11.53)
Social cognition						
MSCEIT	45.17 (10.79)	46.8 (10.61)	44.51 (10.34)	45.62 (10.82)	44.14 (10.56)	43.59 (10.27)
MCCB total score	40.19 (10.62)	43.20 (10.99)	43.94 (11.59)	40.36 (10.09)	41.36 (10.25)	43.13 (11.92)
Digit Span Test^a^	16.29 (4.27)	17.71 (4.89)	17.06 (4.35)	15.36 (5.00)	15.90 (4.66)	15.4 (6.06)
WCST no. of categories^a^	2.09 (2.11)	2.72 (2.20)	2.49 (2.14)	2.19 (2.03)	1.86 (1.98)	1.85 (1.97)
Clinical Symptoms						
PANSS total score	59.77 (12.44)	57.96 (13.23)	58.47 (15.08)	61.62 (13.43)	60.92 (14.97)	59.54 (14.73)
PANSS positive score	12.12 (4.76)	11.86 (4.92)	11.85 (5.19)	12.39 (5.08)	12.51 (5.26)	11.83 (5.00)
PANSS negative score	17.02 (5.17)	16.31 (5.22)	16.94 (6.09)	17.72 (6.10)	17.83 (6.34)	18.02 (6.42)
Functional Outcome						
UPSA	41.39 (9.41)	42.56 (10.84)	42.80 (10.31)	38.36 (9.02)	39.74 (10.78)	41.84 (10.91)
NOSIE						
Total positive factor	78.38 (12.16)	79.31 (12.81)	78.40 (11.79)	73.42 (14.46)	74.94 (14.39)	74.77 (12.90)
Total negative factor	24.13 (14.26)	20.48 (14.01)	20.66 (14.67)	27.43 (14.16)	26.00 (15.78)	22.24 (13.83)
Total score	182.26 (23.21)	187.21 (23.55)	186.22 (23.25)	173.39 (25.17)	177.17 (26.69)	180.48 (24.11)
Self-esteem	27.01 (4.78)	28.78 (3.71)	28.64 (3.66)	26.79 (5.26)	28.34 (4.12)	28.51 (3.70)

CCRT, Computerized Cognitive Remediation Therapy; MCCB, MATRICS Consensus Cognitive Battery; HVLT-R, Hopkins Verbal Learning Test-Revised; BVMT-R, Brief Visuospatial Memory Test-Revised; MSCEIT, Mayer-Salovery-Caruso Emotional Intelligence Test; WCST, Wisconsin Card Sorting Test; PANSS, Positive and Negative Syndrome Scale; UPSA, UCSD Performance-Based Skills Assessment; NOSIE, Nurse's Observation Scale for Inpatient Evaluation.

a The scales were not included in MCCB.

### Is there a treatment effect on the primary and secondary outcomes?

Table 3 shows the results of the formal analyses of the cognitive outcomes, clinical symptoms and functional outcomes. The primary outcome, MCCB total score, showed a significant advantage to CCRT. In addition, five of the secondary cognitive outcomes (Spatial Span, HVLT-R, MSCEIT, Digit Span Test and WCST) also showed a CCRT group advantage.

**Table 3.** Results of the mixed models analyses

Cognitive outcome	Interaction test	Group effect (excluding non-significant interaction)	Estimated advantage to CCRT (no. of points on the scale)/95% CI	Effect size (95% CI)
MCCB				
Speed of processing				
Category Fluency Test (animal)	*F*_(1,228)_ = 0.08; *p* = 0.77	*F*_(1,272)_ = 1.25; *p* = 0.26	1.09 (−0.82 to 3.00)	0.13 (−0.09 to 0.34)
Trail Making Test, Part A	*F*_(1,210)_ = 0.07; *p* = 0.79	*F*_(1,258)_ = 0.84; *p* = 0.36	−1.04 (−3.25 to 1.18)	−0.11 (−0.33 to 0.12)
Symbol Coding Test	*F*_(1,215)_ = 0.81; *p* = 0.37	*F*_(1,263)_ = 1.72; *p* = 0.19	0.92 (−0.46 to 2.31)	0.15 (−0.07 to 0.37)
Attention/Vigilance				
Continuous Performance Test	*F*_(1,205)_ = 1.20; *p* = 0.27	*F*_(1,266)_ = 0.35; *p* = 0.55	0.56 (−1.29 to 2.41)	0.07 (−0.16 to 0.31)
Working memory				
Digit Sequencing Test	*F*_(1,218)_ = 2.94; *p* = 0.09	*F*_(1,266)_ = 0.57; *p* = 0.45	0.64 (−1.03 to 2.30)	0.08 (−0.13 to 0.30)
Spatial Span Test	*F*_(1,211)_ = 0.32; *p* = 0.57	***F*_(1_**_,**268)**_ **= 9.36; *p* = 0.00**	**3.34 (1.19–5.50)**	**0.32 (0.11–0.53)**
Verbal learning				
HVLT-R	*F*_(1,217)_ = 0.00; *p* = 0.98	***F*_(1_**_,**245)**_ **= 4.13; *p* = 0.04**	**2.17 (0.07–4.28)**	**0.22 (0.01–0.43)**
Visual learning				
BVMT-R	*F*_(1,211)_ = 1.80; *p* = 0.18	*F*_(1,261)_ = 1.84; *p* = 0.18	1.25 (−0.56 to 3.06)	0.15 (−0.07 to 0.38)
Reasoning and problem solving				
Mazes Test	*F*_(1,222)_ = 0.02; *p* = 0.89	*F*_(1,252)_ = 1.51; *p* = 0.22	1.28 (−0.77 to 3.34)	0.14 (−0.08 to 0.36)
Social cognition				
MSCEIT	*F*_(1,218)_ = 2.01; *p* = 0.16	***F*_(1_**_,**262)**_ **= 4.55; *p* = 0.03**	**2.19 (0.17–4.20)**	**0.24 (0.02–0.47)**
MCCB total score	*F*_(1,212)_ = 0.04; *p* = 0.84	***F*_(1_**_,**258)**_ **= 5.62; *p* = 0.02**	**1.73 (0.29–3.17)**	**0.27 (0.04–0.49)**
Digit Span Test^a^	*F*_(1,165)_ = 0.18; *p* = 0.68	***F*_(1_**_,**212)**_ **= 8.84; *p* = 0.00**	**1.33 (0.45–2.21)**	**0.38 (0.13–0.63)**
WCST no. of categories^a^	*F*_(1,212)_ = 1.05; *p* = 0.31	***F*_(1_**_,**257)**_ **= 13.5; *p* = 0.00**	**0.73 (0.34–1.12)**	**0.42 (0.20–0.65)**
Clinical Symptoms				
PANSS total score	*F*_(1,235)_ = 0.39; *p* = 0.53	*F*_(1,258)_ = 1.25; *p* = 0.26	−1.25 (−3.46 to 0.95)	−0.12 (−0.32 to 0.09)
PANSS positive score	*F*_(1,243)_ = 1.07; *p* = 0.30	*F*_(1,262)_ = 0.55; *p* = 0.46	−0.28 (−1.03 to 0.46)	−0.08 (−0.28 to 0.13)
PANSS negative score	*F*_(1,233)_ = 0.09; *p* = 0.77	***F*_(1_**_,**258)**_ **= 4.82; *p* = 0.03**	**−0.88 (−1.67 to −0.09)**	**−0.22 (−0.42 to −0.02)**
Functional Outcome				
UPSA	*F*_(1,111)_ = 0.29; *p* = 0.59	*F*_(1,118)_ = 0.05; *p* = 0.83	0.29 (−2.36 to 2.94)	0.04 (−0.32 to 0.40)
NOSIE				
Total positive factor	*F*_(1,212)_ = 0.15; *p* = 0.70	*F*_(1,241)_ = 0.11; *p* = 0.74	0.36 (−1.81 to 2.54)	0.04 (−0.19 to 0.27)
Total negative factor	***F*_(1_**_,**202)**_ **= 4.21; *p* = 0.04**	*F*_(1,230)_ = 1.15; *p* = 0.29	−1.42 (−4.02 to 1.19)	−0.13 (−0.36 to 0.11)
Total score	*F*_(1,200)_ = 2.30; *p* = 0.13	*F*_(1,233)_ = 1.57; *p* = 0.21	2.77 (−1.59 to 7.12)	0.15 (−0.09 to 0.38)
Self-esteem	*F*_(1,247)_ = 0.51; *p* = 0.48	*F*_(1,262)_ = 0.28; *p* = 0.59	0.22 (−0.60 to 1.04)	0.06 (−0.16 to 0.28)

HVLT-R, Hopkins Verbal Learning Test-Revised; BVMT-R, Brief Visuospatial Memory Test-Revised; MSCEIT, Mayer-Salovery-Caruso Emotional Intelligence Test; WCST, Wisconsin Card Sorting Test; PANSS, Positive and Negative Syndrome Scale; UPSA, UCSD Performance-Based Skills Assessment; NOSIS, Nurse's Observation Scale for Inpatient Evaluation.

a The scales were not included in MCCB. Bold indicate statistically significant effects (*p* < 0.05).

For the secondary symptom and functional capacity outcomes far fewer advantages were found. There was a significant effect on the PANSS negative symptom score but none for other symptom measures or functional capacity. However, one significant interaction emerged from the NOSIE, which was opposite to our hypothesis – the Active control group decreased more on the negative factors at follow-up [estimated decrease of 4.63 points for Active control; 95% confidence interval (CI) 1.91–7.35; *p* = 0.001 after Bonferroni correction], while no change was found in the CCRT group (estimated increase of 0.66 points; 95% CI −1.68 to 3.00; *p* > 0.05).

### Does cognitive change mediate functional outcome?

No significant interactions emerged in the ANCOVA analyses and, after removing them, there were significant overall effects of cognitive change on both functional capacity and symptoms. An improvement in the primary outcome (MCCB total score) was marginally associated with a significant improvement in UPSA (*F*_1,108_ = 3.01; *p* = 0.09). However, an improvement in the WCST was associated with a significant improvement in functional capacity as measured by UPSA (*F*_1,108_ = 4.15, *p* = 0.04; estimated improvement was 0.85 points, 95% CI 0.02–1.67) and an improvement in Digit Span was accompanied by a decrease in PANSS positive symptoms (*F*_1,179_ = 5.62, *p* = 0.02; estimated improvement was 0.21 points per extra unit decrease in Digit Span Test, 95% CI 0.03–0.38) (see online Supplementary Table S2 for details).

### Moderators of CCRT benefits

#### Does MCCB baseline total score affect cognitive benefit?

For post-treatment outcome the MCCB baseline total score was negatively correlated with cognitive benefit in both groups (CCRT: *r* = −0.25, df = 170, *p* = 0.001; Active control: *r* = −0.28, df = 87, *p* = 0.009, respectively). After controlling for age, these correlations remained significant (CCRT group, *r* = −0.26, df = 167, *p* = 0.001; Active control group, *r* = −0.282, df = 84, *p* = 0.009). Although baseline cognition was related to sustained cognitive benefit of cognitive remediation (baseline – follow-up), it did not reach traditional statistical significance in the CCRT group (*r* = −0.14, df = 143, *p* = 0.088) and was not significant in the Active control group (*r* = −0.12, df = 61, *p* = 0.344), even after controlling for age (*r* = −0.15, df = 140, *p* = 0.075 and *r* = −0.13, df = 58, *p* = 0.322, respectively).

**Fig. 2.**
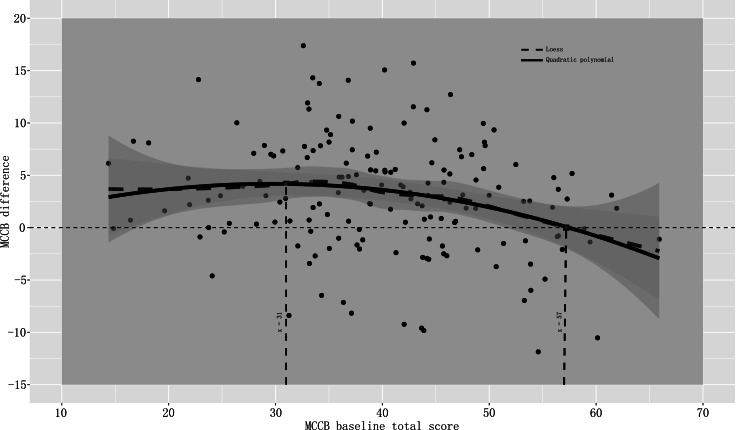
Effects of MCCB baseline total score on CCRT cognitive benefit. *Note*: The baseline total score 31 was the inflection point of polynomial fitting and represented the high point of effect; 57 was the intersection point with zero, and represented the no effect of CCRT on cognitive function.

Figure 2 shows the inflection point of polynomial fitting for the baseline total score. Participants with an MCCB baseline total score around 31 seemed to receive the most cognitive improvement from CCRT; 57 was the intersection point with zero, indicating that the CCRT effect disappears when MCCB baseline total score was more than 57.

#### Do younger and older participants show similar CCRT benefits?

There was no group by age interaction on age, education, antipsychotic agents or cognitive function at baseline among four age subgroups and no group by age interactions on the primary and secondary outcomes (online Supplementary Table S3). The GLM analyses assessed additional fixed effects for age group in exploratory analyses (see online Supplementary Table S4), and after removal of the non-significant interactions, we observed group by age interactions on Spatial Span (*F*_1,271_ = 8.46, *p* = 0.004), and Symbol Coding (*F*_1,269_ = 3.99, *p* = 0.047) with the relevant baseline scores as the covariates. Further analyses revealed that for the younger group, CCRT produced a significant benefit on Symbol Coding (*F*_1,52_ = 5.78, *p* = 0.020; estimated increase 3.71 points, 95% CI 0.61–6.81) at both post-treatment and follow-up but not for the older group (*F*_1,208_ = 0.07, *p* = 0.798; estimated increase 0.20 points, 95% CI −1.33 to 1.72). While, for the older group, CCRT produced a significant benefit on Spatial Span (*F*_1,211_ = 15.73, *p* < 0.001; estimated increase 5.04 points, 95% CI 2.53–7.54) at two time points but not for the younger group (*F*_1,50_ = 1.21, *p* = 0.277; estimated increase −2.03 points, 95% CI −5.74 to 1.68). In addition, there was a significant group by age by time interaction on MSCEIT (*F*_1,219_ = 5.42, *p* = 0.021). Simple effect analyses showed that for the older group CCRT produced a significant benefit on MSCEIT at post-treatment (*F*_1,206_ = 9.11, *p* = 0.003; estimated increase 3.6 points, 95% CI 1.28–6.1) but not at follow-up (*F*_1,165_ = 0.00, *p* = 0.989; estimated increase 0.02 points, 95% CI −2.90 to 2.94). However, for the younger group there was no effect of CCRT on MSCEIT at either time point (post-treatment *F*_1,49_ = 0.00, *p* = 0.947; estimated increase 0.2 points, 95% CI −5.76 to 6.15; follow-up *F*_1,33_ = 0.98, *p* = 0.330; estimated increase 3.67 points, 95% CI −3.88 to 11.22). There was no age effect on clinical symptoms or functional outcome benefits after CCRT treatment.

## Discussion

### Is CCRT effective?

To the best of our knowledge, this is the first randomized controlled study to explore the efficacy of CCRT treatment and the potential mechanism of improvement in a large sample of chronic and stable inpatients with a diagnosis of schizophrenia in China. As hypothesized, participants demonstrate a benefit of CCRT in the overall MCCB total score and specifically in four cognitive domains: working memory (Spatial Span Test, Digit Span Test), Verbal learning (HVLT-R), social cognition (MSCEIT) and executive function (WCST test). These results replicate other studies and meta-analyses and further suggest that CCRT can provide benefits to patients with cognitive difficulties (Grynszpan *et al*., 2011; Wykes *et al*., 2011). Unlike other studies there was no significant direct effect of cognitive remediation on any functional outcome (Wykes *et al*., 2007*a*, 2007*b*, 2011). There are several potential reasons for this lack of direct effect. We compared CCRT to a group who were receiving high levels of psychosocial activities and rehabilitation programs and these potentially have a positive effect on both cognition and functioning making it harder to see a signal, especially over the relatively short follow-up (Glicksohn and Cohen, 2000; Chen *et al*., 2016). A recent study (Buonocore *et al*., 2018) found that although cognition was stable over time following CR, sustained functional improvements required an additional 12 months of standard rehabilitation. A further explanation is that transfer to sustained functional outcome may require other supportive rehabilitation. We did, however, observe a significant direct effect of cognitive remediation on negative symptoms, which replicates previous studies (Bellucci *et al*., 2003; Twamley *et al*., 2012; Cella *et al*., 2017). CCRT has a relatively lack of face to face interaction with the therapist who can explicitly encourage ‘bridging’ strategies and provide social cues to improve participant's self-esteem, whereas the control group had a lot more group activity with interaction actually encouraged. In contrast to the benefit on negative symptoms, the Active control group improved more on the negative NOSIE factor. This is an informant measure of overt negative behaviors, rather than the, mostly, subjective report from the PANSS. The intensity of therapist contact and group interaction in the Active control group may have contributed to these improved negative behaviors. These are important results because a potential limitation of any computerized cognitive remediation is the lack of social and therapeutic contact. Other studies have highlighted the importance of the therapist and the therapeutic alliance drivers of cognitive and functional improvement (e.g. Rose *et al*., 2008; Huddy *et al*., 2012; Reeder *et al*., 2017; Cella and Wykes, 2019). In this study the CCRT therapist did not support the use of strategies, motivation or reinforcement. They also did not help to develop metacognition which is thought to be a key component for improving transfer from cognitive change to functional development (Wykes and Reeder, 2005; Cella *et al*., 2015). It is possible that limited opportunities to practice skills in the three inpatient settings were also a barrier to functional improvement.

### Does cognitive improvement mediate functional benefit?

Previous reviews linked cognitive ability and functional outcome (Green *et al*., 2000; Reeder *et al*., 2004), so we hypothesized that CCRT might increase cognition and that these improvements contribute to improved functional capacity or symptoms. Consistent with this hypothesis, two cognitive change scores (working memory and executive functioning) respectively show a positive effect on symptoms (PANSS positive score) and functional capacity (UPSA), but these relationships were not specific to CCRT. This replicates a previous finding that executive functioning training rather than perceptual training led to improved functioning (Best *et al*., 2019). Our results are also consistent with findings from both Spaulding and Wykes who showed that executive tasks predicted improved social functioning and symptoms, regardless of whether or not participants had received cognitive remediation (Spaulding *et al*., 1999*a*, 1999*b*; Wykes *et al*., 1999, 2007*a*, 2007*b*; Reeder *et al*., 2006). The subtle effects of cognitive remediation therapeutic models have not been surfaced by this study despite larger numbers.

### Does baseline cognition affect treatment benefit?

One of the important findings of this large study was the ability to assess whether and how baseline cognition might have an impact on treatment outcome. Participants with lower MCCB total score (<57 scores) at baseline are likely to benefit more from CCRT at post-treatment. This result is similar to a previous study in which poorer initial memory performances seemed to predict larger CR improvements (Pillet *et al*., 2015). Similarly Bell *et al*. discovered sustained effects of CR on work outcomes only in those individuals who entered the study with poorer levels of functioning (Bell *et al*., 2014). Although our result needs replication, it does suggest that with scarce health service resources, targeting those with lower baseline scores with the current version of CCRT would achieve the most benefit (see clinical implications).

### Does age affects the cognitive benefits from CCRT?

The overall CCRT benefit (MCCB total score) was independent of age. But for some cognitive domains, the benefits varied between younger and older participants as in other studies. Younger participants improved on verbal processing speed, whereas older participants showed reduced positive symptoms and less decline in self-reported daily functioning. A similar differential pattern of improvements was found in another smaller study (Thomas *et al*., 2017). The advantage shown in the younger participants may be due to increased cognitive plasticity, especially in a basic cognitive function such as speed (Bürki *et al*., 2014). The larger cognitive effects on Spatial Span in the older group may be due to poor spatial working memory at baseline (as shown in online Supplementary Table S3), so the older participants have more room for improvement (Kern *et al*., 2008). The effects on emotion processing in the older group may be because, unlike basic cognitive functions, social cognitions such as emotional management in older patients improve with age (Kern *et al*., 2008).

There is evidence that CCRT facilitates benefits in both younger and older participants but in different domains. This makes the assessment of both moderators and mediators complex when samples have a wide age range and will require an even larger sample size to assess effects.

## Clinical and development implications

The program improves cognition and negative symptoms but has limitations on functioning improvement. Currently the program does not offer many cognitive benefits if the baseline score is more than 57. With limited resources focusing on patients with an MCCB baseline total score around 31 who showed the most cognitive improvement from CCRT might be the most efficient way to use those resources. It is still unclear, however, whether the treatment should only be recommended if some cognitive areas show deterioration (despite the total score being above a *T*-score of 40). The study took part in hospitals with relatively little opportunity for transfer of cognitive benefit to functioning and the therapists were also not as involved throughout therapy. The next step may be to add transfer tasks to CCRT, to involve therapists in emphasizing metacognitive processes and considering providing further opportunities for developing transferable skills (see Reeder *et al*., 2018). In addition, CCRT could add more challenging tasks that might support cognitive benefit in those with better baseline cognitive function which might then boost cognition.

## Strengths and limitations

This is the largest study of cognitive remediation. It replicated the beneficial cognitive effects which was particularly impressive as we included only those individuals who had a chronic course and experienced some cognitive deficits. But there was only a short follow-up so we may have underestimated the longer-term functioning benefits. Those improvements may develop slowly as opportunities to learn or relearn new skills are offered in the relatively restricted environment in the hospital. Further studies should include environmental supports that would allow the exploration of real life functioning. Age moderates CCRT benefit but only for individual cognitive tests rather than general cognitive function. Although this differential pattern has been found in other studies, the results need replication. Our sample did not include individuals living in the community which limits generalization. However, even in those with longer illness and poorer recovery trajectories, CCRT was beneficial and age was not a barrier to improvement.

## Conclusions

To our knowledge, this is the largest cognitive remediation study which allowed us to explore mediation and moderation effects as well as efficacy. This form of cognitive remediation shows efficacy for cognition and negative symptoms, and evidence that those with poorer cognition show more cognitive improvement. Age was not a barrier to cognitive improvement although the cognitive domain benefits differed between age groups. Evidence that people with poorer cognition show more benefit needs further investigation as it is unclear whether achieving a large cognitive change is a barrier to functional improvement in this therapy. Identifying factors that influence cognitive benefit is the beginning of providing personalization and tailored care in clinical cognitive therapy applications.

## Supplementary material

For supplementary material accompanying this paper visit http://dx.doi.org/10.1017/S0033291719001594. click here to view supplementary material
