# Supplementary material for: Who will benefit from computerized cognitive remediation therapy? Evidence from a multisite randomized controlled study in schizophrenia
Source: Psychol Med. 2019 Jul 12;50(10):1633–43. doi: 10.1017/S0033291719001594 (PMC7408576; doi:10.1017/S0033291719001594)
Supplement: Supplementary file 1 [file S0033291719001594sup.zip › S0033291719001594sup001.docx]

**Supplementary tables**

**Table 1.** Demographic, clinical and cognitive variables of the two groups about taken off

| Characteristics | CCRT group | | Active control group | | CCRT | | Active control | |
| --- | --- | --- | --- | --- | --- | --- | --- | --- |
|  | Dropout  (n = 26) | None  (n = 170) | Dropout  (n = 28) | None  (n = 87) | *χ^2^* | *P* | *χ^2^* | *P* |
| Sex (male/female) | 12:14 | 110:60 | 15:13 | 54:33 | 3.303 | 0.069 | 0.637 | 0.425 |
| Marriage (yes/no) | 6:20 | 42:128 | 10:18 | 18:69 | 0.032 | 0.857 | 2.596 | 0.107 |
| Receiving SGA | 1:11 | 30:136 | 3:13 | 13:72 | 0.738 | 0.390 | 0.121 | 0.728 |
|  |  |  |  |  | Group × Dropout | | Dropout | |
|  |  |  |  |  | *F* | *P* | *F* | *P* |
| Mean age (SD), year | 42.50±8.42 | 46.08±8.64 | 42.46±8.03 | 44.82±8.36 | 0.227 | 0.634 | 5.322 | 0.022 |
| Mean education years (SD), year | 13.04±3.09 | 11.54±2.73 | 12.39±3.02 | 11.69±2.62 | 0.906 | 0.342 | 6.955 | 0.009 |
| Dose of chlorpromazine equivalents | 474.94±221.67 | 402.51±262.92 | 407.81±218.27 | 355.54±222 | 0.041 | 0.840 | 1.553 | 0.214 |
| MCCB total score | 40.90±9.66 | 40.08±10.78 | 39.60±10.68 | 40.57±9.97 | 0.303 | 0.582 | 0.002 | 0.963 |
| Clinical Symptoms |  |  |  |  |  |  |  |  |
| PANSS total score | 59.00±14.16 | 59.82±12.35 | 58.44±13.74 | 62.21±13.38 | 0.324 | 0.570 | 0.784 | 0.377 |
| PANSS positive score | 12.83±4.99 | 12.07±4.76 | 9.88±3.70 | 12.86±5.18 | 3.663 | 0.057 | 1.279 | 0.259 |
| PANSS negative score | 15.75±6.00 | 17.11±5.11 | 16.88±6.77 | 17.87±6.00 | 0.026 | 0.872 | 1.108 | 0.293 |
| UPSA | 43.44±11.17 | 41.08±9.14 | 47.78±5.45 | 37.51±8.83 | 1.644 | 0.202 | 4.190 | 0.043 |
| NOSIE |  |  |  |  |  |  |  |  |
| Total positive factor | 73.73±12.62 | 78.70±12.11 | 74.63±14.46 | 73.19±14.54 | 1.403 | 0.237 | 0.427 | 0.514 |
| Total negative factor | 24.00±13.36 | 24.14±14.35 | 27.06±15.23 | 27.50±14.04 | 0.002 | 0.960 | 0.010 | 0.922 |
| Total score | 177.73±23.60 | 182.57±23.22 | 175.56±27.74 | 172.96±24.79 | 0.559 | 0.455 | 0.051 | 0.822 |

CCRT, Computerized Cognitive Remediation Therapy; MCCB, MATRICS Consensus Cognitive Battery; PANSS, Positive and Negative Syndrome Scale; UPSA, UCSD Performance-Based Skills Assessment; NOSIE, Nurse’s Observation Scale for Inpatient Evaluation.

**Table 2.** Effects of cognitive change on functional outcomes

|  | MCCB total score | Spatial Span Test | HVLT-R | MSCEIT | WCST | Digit Span Test |
| --- | --- | --- | --- | --- | --- | --- |
| PANSS total score |  |  |  |  |  |  |
| Interaction | F(1,223) = 2.70; p = 0.10 | F(1,228) = 1.35; p = 0.25 | F(1,227) = 0.48; p = 0.49 | F(1,224) = 0.09; p = 0.76 | F(1,216) = 0.03; p = 0.87 | F(1,178)= 1.17; p = 0.28 |
| Main effect | F(1,224) = 0.45; p = 0.50 | F(1,229) = 0.02; p = 0.89 | F(1,228) = 1.38;p = 0.24 | F(1,225) = 0.98; p = 0.32 | F(1,217) = 0.37; p = 0.54 | F(1,179) = 1.92; p = 0.17 |
| PANSS positive score |  |  |  |  |  |  |
| Interaction | F(1,223) = 0.14; p = 0.71 | F(1,228) = 0.69; p = 0.41 | F(1,227) = 0.24; p = 0.62 | F(1,224) = 0.01; p = 0.93 | F(1,216) = 1.05; p = 0.31 | F(1,178) = 0.99; p = 0.32 |
| Main effect | F(1,224) = 1.43; p = 0.23 | F(1,229) = 0.38; p = 0.54 | F(1,228) = 1.49; p = 0.22 | F(1,225) = 1.02; p = 0.31 | F(1,217) = 1.83; p = 0.18 | **F(1,179) = 5.62; p = 0.02** |
| PANSS negative score |  |  |  |  |  |  |
| Interaction | F(1,223) = 2.59; p = 0.11 | F(1,228) = 0.63; p = 0.43 | F(1,227) = 0.50; p = 0.48 | F(1,224) = 0.96; p = 0.33 | F(1,216) = 0.16; p = 0.69 | F(1,178) = 2.23; p = 0.14 |
| Main effect | F(1,224) = 0.12; p = 0.73 | F(1,229) = 0.31; p = 0.58 | F(1,228) = 0.96; p = 0.33 | F(1,225) = 0.83; p = 0.36 | F(1,217) = 0.33; p = 0.57 | F(1,179) = 0.11; p = 0.74 |
| UPSA |  |  |  |  |  |  |
| Interaction | F(1,107) = 0.14; p = 0.70 | F(1,107) = 2.55; p = 0.11 | F(1,107) = 0.19; p = 0.66 | F(1,107) = 0.02; p = 0.89 | F(1,107) = 0.89; p = 0.35 | F(1,105) = 0.01; p = 0.94 |
| Main effect | F(1,108) = 3.01; p = 0.09 | F(1,108) = 3.70; p = 0.06 | F(1,108) = 0.96; p = 0.33 | F(1,108) = 1.67; p = 0.20 | **F(1,108) = 4.15; p = 0.04** | F(1,106) = 0.00; p = 0.99 |
| NOSIE positive factors |  |  |  |  |  |  |
| Interaction | F(1,178) = 0.60; p = 0.44 | F(1,183) = 0.61; p = 0.44 | F(1,182) = 1.91; p = 0.17 | F(1,179) = 0.00; p = 0.97 | F(1,173) = 1.27; p = 0.26 | F(1,147) = 0.02; p = 0.89 |
| Main effect | F(1,179) = 0.32; p = 0.58 | F(1,184) = 2.75; p = 0.10 | F(1,183) = 0.13; p = 0.72 | F(1,180) = 0.42; p = 0.52 | F(1,174) = 0.33; p = 0.57 | F(1,148) = 0.00; p = 0.95 |
| NOSIE negative factors |  |  |  |  |  |  |
| Interaction | F(1,173) = 0.15; p = 0.70 | F(1,178) = 1.98; p = 0.16 | F(1,177) = 0.03; p = 0.85 | F(1,174) = 0.10; p = 0.75 | F(1,167) = 0.21; p = 0.65 | F(1,141) = 0.57; p = 0.45 |
| Main effect | F(1,174) = 0.00; p = 0.97 | F(1,179) = 0.10; p = 0.76 | F(1,178) = 0.06; p = 0.81 | F(1,175) = 0.37; p = 0.54 | F(1,168) = 0.00; p = 0.96 | F(1,142) = 0.00; p = 0.98 |
| NOSIE total score |  |  |  |  |  |  |
| Interaction | F(1,168) = 0.00; p = 0.99 | F(1,173) = 1.61; p = 0.21 | F(1,172) = 0.33; p = 0.56 | F(1,169) = 0.08; p = 0.78 | F(1,163) = 0.81; p = 0.37 | F(1,140) = 0.41; p = 0.53 |
| Main effect | F(1,169) = 0.01; p = 0.93 | F(1,174) = 1.04; p = 0.31 | F(1,173) = 0.14; p = 0.71 | F(1,170) = 0.50; p = 0.48 | F(1,164) = 0.02; p = 0.88 | F(1,141) = 0.00; p = 0.99 |
| Self-esteem |  |  |  |  |  |  |
| Interaction | F(1,219) = 0.12; p = 0.73 | F(1,224) = 0.10; p = 0.75 | F(1,223) = 0.50; p = 0.48 | F(1,221) = 0.37; p = 0.55 | F(1,213) = 0.69; p = 0.41 | F(1,177) = 2.85; p = 0.09 |
| Main effect | F(1,220) = 0.11; p = 0.74 | F(1,225) = 0.04; p = 0.85 | F(1,224) = 0.41; p = 0.52 | F(1,222) = 1.47; p = 0.23 | F(1,214) = 0.08; p = 0.77 | F(1,178) = 0.44; p = 0.51 |

MCCB, MATRICS Consensus Cognitive Battery; HVLT-R, Hopkins Verbal Learning Test-Revised; MSCEIT, Mayer-Salovery-Caruso Emotional Intelligence Test; WCST, Wisconsin Card Sorting Test; PANSS, Positive and Negative Syndrome Scale; UPSA, UCSD Performance-Based Skills Assessment; NOSIE, Nurse’s Observation Scale for Inpatient Evaluation.

Table 3

**Table 3.** Demographic, clinical and cognitive variables of the two groups by age

| Characteristics | CCRT group | | Active control group | | CCRT | | Active control | |
| --- | --- | --- | --- | --- | --- | --- | --- | --- |
|  | young  (n =40) | old  (n = 156) | young  (n = 29) | old  (n = 86) | *χ^2^* | *P* | *χ^2^* | *P* |
| Sex (male/female) | 19:21 | 103:53 | 15:14 | 54:32 | 4.649 | 0.031 | 1.107 | 0.293 |
| Marriage (yes/no) | 5:35 | 43:113 | 4:25 | 24:62 | 3.907 | 0.048 | 2.345 | 0.126 |
| Psychiatric medication (typical/Atypical and both) | 8:27 | 23:220 | 5:20 | 11:65 | 0.897 | 0.344 | 0.431 | 0.512 |
|  |  |  |  |  | Group × Age interaction | | Age group | |
|  |  |  |  |  | *F* | *P* | *F* | *P* |
| Mean age (SD), year | 32.75±6.57 | 48.90±5.5 | 33.41±6.09 | 47.90±5.19 | 1.133 | 0.288 | 382.975 | 0.000 |
| Mean education years (SD), year | 13.55±2.67 | 11.28±2.67 | 13.14±2.59 | 11.43±2.65 | 0.587 | 0.444 | 28.971 | 0.000 |
| Dose of chlorpromazine equivalents | 471.22±252.71 | 391.77±260.77 | 407.98±197.55 | 349.22±227.81 | 0.079 | 0.778 | 3.546 | 0.061 |
| Cognitive Outcome |  |  |  |  |  |  |  |  |
| Symbol Coding Test | 38.24±8.98 | 41.62±9.26 | 40.53±8.94 | 40.29±9.00 | 1.965 | 0.162 | 1.471 | 0.226 |
| Digit Sequencing Test | 46.05±10.76 | 44.94±9.88 | 46.77±12.83 | 42.30±9.27 | 1.388 | 0.240 | 3.834 | 0.051 |
| Spatial Span Test | 46.81±8.81 | 42.49±12.12 | 45.83±11.04 | 40.46±12.99 | 0.099 | 0.753 | 8.364 | 0.004 |
| ^*^Digit Span Test | 19.75±3.97 | 15.38±3.87 | 19.92±4.96 | 13.84±4.01 | 2.024 | 0.156 | 76.115 | 0.000 |
| MSCEIT | 47.59±11.31 | 44.58±10.61 | 49.05±12.29 | 44.47±10.1 | 0.263 | 0.608 | 6.221 | 0.013 |
| MCCB total score | 39.89±10.18 | 40.27±10.75 | 42.99±12.72 | 39.52±9.01 | 1.695 | 0.194 | 1.095 | 0.296 |
| NOSIE |  |  |  |  |  |  |  |  |
| Total negative factor | 24.50±13.88 | 24.04±14.39 | 28.08±12.47 | 27.21±14.76 | 0.009 | 0.922 | 0.096 | 0.757 |
| Total score | 181.97±20.85 | 182.33±23.83 | 173.25±21.48 | 173.44±26.40 | 0.001 | 0.981 | 0.006 | 0.940 |

CCRT, Computerized Cognitive Remediation Therapy; MCCB, MATRICS Consensus Cognitive Battery; PANSS, Positive and Negative Syndrome Scale; UPSA, UCSD Performance-Based Skills Assessment; NOSIE, Nurse’s Observation Scale for Inpatient Evaluation.

**Table 4.** Results of the mixed models analyses on age group

|  | Group×time×age group Interaction | Group×time interaction  (excluding non-significant interaction) | Time×age group interaction  (excluding non-significant interaction) | Group×age group interaction  (excluding non-significant interaction) |
| --- | --- | --- | --- | --- |
| Cognitive Outcome |  |  |  |  |
| Speed of processing |  |  |  |  |
| Category Fluency Test (Animal) | F(1,230) = 0.20; p = 0.66 | F(1,227) = 0.11; p = 0.74 | F(1,234) = 0.03; p = 0.86 | F(1,276) = 1.05; p = 0.31 |
| Trail Making Test, Part A | F(1,217) = 0.81; p = 0.37 | F(1,210) = 0.05; p = 0.82 | F(1,217) = 0.40; p = 0.53 | F(1,263) = 0.20; p = 0.66 |
| Symbol Coding Test | F(1,222) = 0.12; p = 0.73 | F(1,216) = 0.65; p = 0.42 | F(1,224) = 2.13; p = 0.15 | **F(1,269) = 3.99; p = 0.05** |
| Attention/Vigilance |  |  |  |  |
| Continuous Performance Test | F(1,206) = 2.06; p = 0.15 | F(1,203) = 1.14; p = 0.29 | F(1,207) = 0.14; p = 0.71 | F(1,268) = 0.02; p = 0.89 |
| Working memory |  |  |  |  |
| *Digit Span Test | F(1,163) = 0.71; p = 0.40 | F(1,164) = 0.15; p = 0.70 | F(1,167) = 0.01; p = 0.94 | F(1,212) = 1.38; p = 0.24 |
| Digit Sequencing Test | F(1,220) = 0.71; p = 0.40 | F(1,217) = 2.93; p = 0.09 | F(1,224) = 0.03; p = 0.86 | F(1,270) = 0.31; p = 0.58 |
| Spatial Span Test | F(1,214) = 0.11; p = 0.74 | F(1,209) = 0.34; p = 0.56 | F(1,215) = 0.11; p = 0.74 | **F(1,271) = 8.46; p = 0.00** |
| Verbal learning |  |  |  |  |
| HVLT-R | F(1,223) = 0.33; p = 0.57 | F(1,217) = 0.00; p = 0.98 | F(1,224) = 0.86; p = 0.36 | F(1,252) = 0.58; p = 0.45 |
| Visual learning |  |  |  |  |
| BVMT-R | F(1,214) = 0.20; p = 0.66 | F(1,208) = 1.52; p = 0.22 | F(1,215) = 2.32; p = 0.13 | F(1,264) = 0.51; p = 0.48 |
| Reasoning and problem solving |  |  |  |  |
| Mazes Test | F(1,230) = 0.15; p = 0.70 | F(1,222) = 0.00; p = 0.95 | F(1,231) = 0.44; p = 0.51 | F(1,259) = 1.51; p = 0.22 |
| *WCST | F(1,214) = 1.32; p = 0.25 | F(1,211) = 1.17; p = 0.28 | F(1,216) = 1.04; p = 0.31 | F(1,261) = 0.40; p = 0.53 |
| Social cognition |  |  |  |  |
| MSCEIT: Managing Emotions | **F(1,219) = 5.42; p = 0.02** | F(1,219) = 0.05; p = 0.82 | F(1,219) = 1.40; p = 0.24 | F(1,263) = 0.01; p = 0.91 |
| MCCB global cognition | F(1,211) = 0.84; p = 0.36 | F(1,211) = 0.05; p = 0.83 | F(1,212) = 0.45; p = 0.51 | F(1,259) = 0.47; p = 0.50 |
| Clinical Symptoms |  |  |  |  |
| PANSS Total Scores | F(1,237) = 0.29; p = 0.59 | F(1,233) = 0.31; p = 0.58 | F(1,236) = 0.25; p = 0.62 | F(1,259) = 2.01; p = 0.16 |
| PANSS Positive Scores | F(1,247) = 0.74; p = 0.39 | F(1,242) = 0.96; p = 0.33 | F(1,247) = 0.00; p = 0.98 | F(1,266) = 1.14; p = 0.29 |
| PANSS Negative Scores | F(1,234) = 0.63; p = 0.43 | F(1,232) = 0.07; p = 0.80 | F(1,234) = 0.12; p = 0.73 | F(1,258) = 0.09; p = 0.77 |
| Functional Outcome |  |  |  |  |
| UPSA | F(1,106) = 0.07; p = 0.80 | F(1,108) = 0.36; p = 0.55 | F(1,113) = 1.26; p = 0.27 | F(1,113) = 0.15; p = 0.70 |
| NOSIE |  |  |  |  |
| Total Positive Factors | F(1,220) = 0.54; p = 0.46 | F(1,210) = 0.08; p = 0.78 | **F(1,222) = 6.39; p = 0.01** | F(1,266) = 2.67; p = 0.10 |
| Total Negative Factors | F(1,211) = 0.12; p = 0.73 | F(1,200) = 3.44; p = 0.07 | **F(1,214) = 5.15; p = 0.02** | F(1,258) = 3.23; p = 0.07 |
| Total Patient Assets | F(1,208) = 0.00; p = 0.96 | F(1,198) = 1.74; p = 0.19 | **F(1,212) = 7.38; p = 0.01** | F(1,255) = 3.59; p = 0.06 |
| Self-esteem | F(1,253) = 0.61; p = 0.44 | F(1,246) = 0.57; p = 0.45 | F(1,254) = 0.32; p = 0.57 | F(1,268) = 1.24; p = 0.27 |

*Note*: * the scales were not included in MCCB; HVLT-R, Hopkins Verbal Learning Test-Revised; BVMT-R, Brief Visuospatial Memory Test-Revised; WCST, Wisconsin Card Sorting Test; MSCEIT, Mayer-Salovery-Caruso Emotional Intelligence Test; PANSS, Positive and Negative Syndrome Scale; UPSA, UCSD Performance-Based Skills Assessment; NOSIE, Nurse’s Observation Scale for Inpatient Evaluation.
