# Supplementary material for: Who will benefit from computerized cognitive remediation therapy? Evidence from a multisite randomized controlled study in schizophrenia
Source: Psychol Med. 2019 Jul 12;50(10):1633–43. doi: 10.1017/S0033291719001594 (PMC7408576; doi:10.1017/S0033291719001594)
Supplement: Supplementary file 1 [file S0033291719001594sup.zip › S0033291719001594sup002.docx]

**Therapy**

**Cognitive remediation**

The computer software adopted was Computerized Cognitive Remediation Therapy System (CCRTS) which was developed by the research team, specifically based on the principals of cognitive remediation therapy such as errorless learning, scaffolding, positive feedback, individualized therapy, efficient information processing strategies, and so on derived from Wykes and Reeder (2005) (Wykes & Reader,2005). CCRTS consists of 30 different cognitive remediation exercises to improve different cognitive skills and a database to store participant’s performance. Most exercises focused on cognitive flexibility, working memory, planning and social function, especially emotion management. These domains were selected on the basis of the three treatment targets (cognitive flexibility, working memory, planning) of cognitive remediation therapy which had been demonstrated to improve the cognitive impairments of participants with schizophrenia (Reeder et al.,2004). As social cognition is associated with functional outcomes (Harvey & Penn,2010; Hooker & Park,2002), three social cognitive remediation exercises focused on facial emotion recognition, context emotion estimation and emotional management were also included. Facial emotion recognition requires the participant to identify the correct emotion expression (sad, angry, fearful, surprise, disgust or happy) for one emotional face image presented on the screen and as difficulty level increased, the strength of facial emotion decreased. Context emotion estimation was trained using a short paragraph that describes an emotional event, such as failing an examination, participants were asked to figure out the emotional state of the person and select one correct emotional face. The intensity of emotional events decreased as difficulty level increased gradually. In the emotion management test, participants were asked to judge the potential effect of three different behaviors on improving mood and to choose one, such as when a young woman loses the person she loves.

Each exercise consists of 6-25 tasks with increasing difficulty which changes dynamically as accuracy reaches 80%, so that an errorless learning environment with high levels of positive feedback can be provided. Different exercises are presented in each session to encourage a higher level of participant engagement. CCRTS was refined and its feasibility and tolerability tested in a previous study (Shuping et al.,2010) (CCRTS details are available from the corresponding author).

CCRT treatment is provided as 4-5 45 minute sessions per week over 12 weeks with a total of 50 sessions. It is supervised by experienced therapists at a ratio of 1 therapist to 4 participants. All therapists had followed a dedicated training program involving theory, observation and supervised practice sessions. The therapy was delivered in groups. The main responsibility of the therapist is to teach the participants to use the CCRT system in the first two weeks, and the subsequent treatment is mainly carried out by the participant alone.

**Active control**

The active control had the same number of therapeutic sessions as the CCRT group. It contained two different activities: playing music (learning to play a fairly easy instrument, e.g. xylophone) and learning to dance, which were generally offered to almost every inpatient in these hospitals. The duration and frequency of therapeutic exposure in the control group were identical to those of the CCRT group. The therapy was also delivered in groups. During each therapy session, the therapist was also actively involved in the music and dancing, which is different from the CCRT group.

**References**

Harvey, P.D., & Penn, D. (2010). Social cognition: the key factor predicting social outcome in people with schizophrenia. *Psychiatry*, 7,41-44.

Hooker, C., & Park, S. (2002). Emotion processing and its relationship to social functioning in schizophrenia patients. *Psychiatry research*, 112,41-50.

Reeder, C., Newton, E., Frangou, S., & Wykes, T. (2004). Which executive skills should we target to affect social functioning and symptom change? A study of a cognitive remediation therapy program. *Schizophrenia bulletin*, 30,87-100.

Shuping, T., Yizhuang, Z., Jian, W., Fude, Y., Guanghui, Z., Yong, C., Jiefeng, C., Nan, C., Hongzhen, F., Biao, H., Lixia, Z., Chongsheng, S., Qinyun, L., Yongchang, L., Wei, Q., Xia, A., Dong, L., Xiaoling, L., Dongfeng, Z., Xiangqun, W., Wenxiang, Q., Zhanjiang, L., & Junhua, G. (2010). Effects of cognitive remediation therapy and computerized cognitive remediation therapy on cognitive deficits in patients with schizophrenia: a randomized controlled study. *CHINESE JOURNAL OF PSYCHIATRY*, 43,140-145.

Wykes, T., & Reader, C. (2005). Cognitive Remediation Therapy for Schizophrenia:Theory & practice. *London: Routledge*, ,pp:315.
